# Mechanical Fatigue Resistance of Piezoelectric PVDF Polymers

**DOI:** 10.3390/mi9100503

**Published:** 2018-10-04

**Authors:** Youn-Hwan Shin, Inki Jung, Hyunchul Park, Jung Joon Pyeon, Jeong Gon Son, Chong Min Koo, Sangtae Kim, Chong-Yun Kang

**Affiliations:** 1 KU-KIST Graduate School of Converging Science and Technology, Korea University, Seoul 02841, Korea; shw9106@kist.re.kr (Y.-H.S.); nasa1011@kist.re.kr (I.J.); 114342@kist.re.kr (J.J.P.); koo@kist.re.kr (C.M.K.); 2 Center for Electronic Materials, Korea Institute of Science and Technology, Seoul 02792, Korea; 3 Materials Architecturing Research Center, Korea Institute of Science and Technology, Seoul 02792, Korea; hpark@kist.re.kr; 4 Photo-Electronic Hybrid Research Center, Korea Institute of Science and Technology, Seoul 02792, Korea; jgson@kist.re.kr

**Keywords:** ferroelectric, PVDF, piezoelectric, mechanical fatigue resistance, remnant polarization

## Abstract

The fatigue resistance of piezoelectric PVDF has been under question in recent years. While some report that a significant degradation occurs after 10^6^ cycles of repeated voltage input, others report that the reported degradation originates from the degraded metal electrodes instead of the piezoelectric PVDF itself. Here, we report the piezoelectric response and remnant polarization of PVDF during 10^7^ cycles of repeated compression and tension, with silver paste-based electrodes to eliminate any electrode effect. After applying repeated tension and compression of 1.8% for 10^7^ times, we do not observe any notable decrease in the output voltage generated by PVDF layers. The results from tension experiments show stable remnant polarization of 5.5 μC/cm^2^, however, the remnant polarization measured after repeated compression exhibits a 7% decrease as opposed to the tensed PVDF. These results suggest a possible anisotropic response to stress direction. The phase analyses by Raman spectroscopy reveals no significant change in the phase content, demonstrating the fatigue resistance of PVDF.

## 1. Introduction

Piezoelectric polymers such as polyvinylidene fluoride (PVDF) have been studied extensively in terms of their piezoelectric properties, with applications ranging from roadway energy harvesting to film-based speakers and biomedical applications [1,2,3]. Some of these applications require excellent mechanical and polarization fatigue resistance. For instance, energy harvesters for roadway applications need to withstand mechanical shocks on the order of 10^8^ times, assuming the daily traffic of 13,000 vehicles and the road’s life expectancy of 10 years [4,5]. The PVDF layers for film speakers require similar resistance against polarization fatigue, assuming the speaker plays 500 Hz audio sound for 56 h [2]. Also, the PVDF-based generator exhibits stable electric output with no degradation after 5 days of in vivo operation (mouse) or 1.512 × 10^8^ times mechanical deformation [3].

However, measuring the fatigue resistance of PVDF remains difficult, since the metal electrodes on flexible PVDF easily deform under repeated strain and affect the measurements. For instance, Zhu et al. reported in 2006 that the remnant and saturation polarization of PVDF copolymered with trifluoroethylene (PVDF-TrFE) decays by 40% when repeated electrical bias up to 10^6^ times is applied [6]. In a recent paper, however, Zhao et al. showed that this decay in polarization originates from partial delamination of metal electrodes during fast bias switching instead of the degradation of PVDF layer itself [7]. In direct application of strain via bending, similar degradation behavior of metal electrodes is expected. For example, Sim et al. showed that polymer-supported metal thin films undergo crack propagation, debonding or delamination, leading to increased resistance under repeated mechanical strain [8,9]. Since the elastic limit of metal thin films falls far below that of flexible polymers, the accumulating plastic deformation on metal thin films inevitably results in the inaccurate assessment of PVDF’s piezoelectric properties under mechanical fatigue.

While PVDF is known for its fatigue resistance against crack propagation [10], understanding the mechanical fatigue resistance against PVDF’s piezoelectric properties remains to be elucidated. In particular, the ferroelectric/piezoelectric properties of PVDF depend strongly on its polymorphs (α-, β-, γ- and δ-phases) [11,12]. Any fatigue-induced phase transformation, as well as mechanical failure, may cause decay in the material’s piezoelectric properties [13,14]. Extensive research effort has been placed on controlling the phase content of PVDF, and various thermodynamic handles including temperature, stress or external electric field are known to affect the phase content. For instance, Sencadas et. al. reported that stretching PVDF induces α- to β-phase transformation [10]. To the best of our best knowledge, however, the phase evolution of poled PVDF under mechanical fatigue and its link to piezoelectric properties have not been studied extensively. Furthermore, no direct fatigue measurements of piezoelectric PVDF by applying repeated strain exists in the literature.

In this work, we assess the fatigue resistance of PVDF as piezoelectric harvesters by measuring the remnant polarization, piezoelectric voltage output, and relative phase contents after repeated mechanical input. To minimize the effects from electrode degradation, we employ commercially available Ag paste-based electrodes with protective sealing. This minimizes the change in the contact area between the electrode and PVDF layers during extended fatigue tests.

## 2. Materials and Methods

### 2.1. PVDF Fatigue Preparation

**Figure 1:**
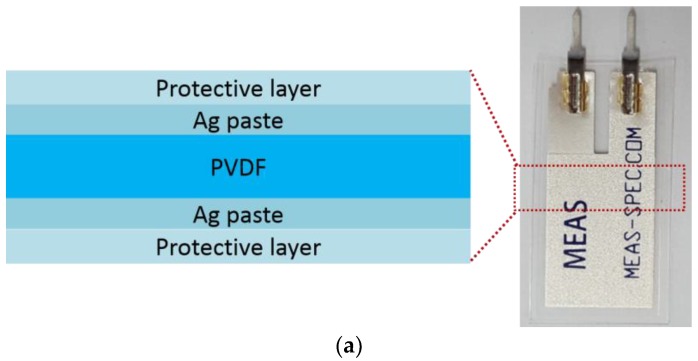

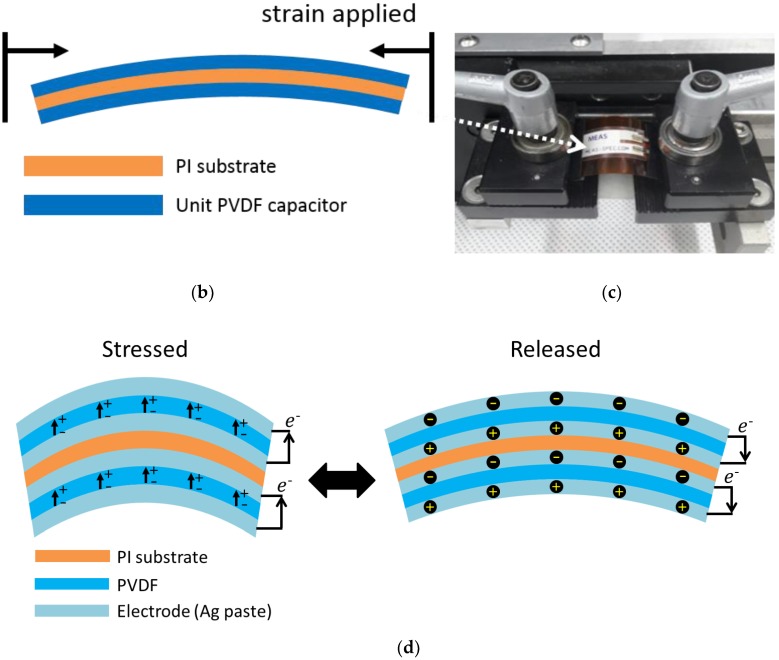
The test setup for piezoelectric PVDF’s fatigue measurement system (**a**) Unit PVDF capacitor with silver paste electrode and sealing layer (**b**) PVDF based piezoelectric generator device with PI substrate (**c**) The homemade servo motor-based bending system with controlled frequency and input displacement. (**d**) The unit PVDF capacitor based bimorph-shaped device with dipole arrangement inside the PVDF and electric flow.

Figure 1 shows the unit PVDF capacitor (Piezo film sensor: DT1-028K, TE connectivity) used in this study and the stress application apparatus. The capacitor consists of 28 μm-thick PVDF layer and Ag paste-based electrodes on both sides (Figure 1a). The entire capacitor is wrapped with a protective polymer coating layer, ensuring that the paste-based electrodes stay intact during repeated bending. The dimensions of the unit capacitor are 16 mm wide, 41 mm long and 40 μm thick, with the measured capacitance of 1.38 nF. The unit capacitors are then attached to 230 μm-thick polyimide (PI) substrates on both sides (Figure 1b). Nickel mesh-based, 30 μm-thick double side tapes (Soluteta, Inc., Hwaseong, Korea) are used to attach the capacitors to the substrates, and the lead wires from the capacitors are protected with conducting carbon tapes to ensure tight attachment during repeated bending tests and minimize background noise.

### 2.2. Fatigue Test Setup and Experimental Measurements

Figure 1c shows the linear motor set up to apply repeated strain to the device. The linear motor is based on a home-made servo motor with 420 rpm (revolution per minute). This is equivalent to 7 Hz bending at 13 mm horizontal displacement. As the motor moved inward, the mechanical stress is transmitted along the longitudinal direction of the unit capacitor. Bending the device generates tensile stress on the top capacitor and compressive stress on the bottom capacitor since the neutral axis lies at the center of the PI substrate. By tracking the change in radii of curvature, we estimate the amount of strain applied and the strain rate. The applied strain on both tensed and compressed PVDFs are 1.8% at the strain rate of 12.7%/s. The amount of strain is ensured to lie within the elastic limit (2.5%) of poled PVDF [15]. Figure 1d depicts the bimorph-shaped device with dipole arrangement inside PVDF and electric charge on the electrode. As we repeat bending and releasing the harvester using the home-made servo motor, it generates piezoelectric charges which can be extracted as an electric circuit.

### 2.3. Electrical Measurements–Voltage Output and Remnant Polarization

The open circuit voltage output is measured with a digital oscilloscope (DPO4014B, Tektronix, Seoul, Korea) during the entire course of bending. The bending is repeated up to 10^7^ times, approximately 17 continuous days at 7 Hz. To accurately capture any decrease in the short-lived voltage peaks, we use the sampling rate of 500 pt/s.

After the completion of the fatigue tests, the Ag paste electrodes and the capping polymer layers are removed with ethanol. On the bare PVDF thin film, we deposit 100 nm-thick Pt electrodes via sputtering. The electrode area is maintained to be 1 × 1 cm^2^ on both sides. The P-E hysteresis loops are measured by a ferroelectric testing system (RT66A, Radiant Technologies, Inc. Precision) connected with a high-voltage amplification interface (AMT-10B10, MATSUSADA Precision Inc., Kusatsu, Japan).

### 2.4. Phase Measurements by Raman Spectroscopy

The fatigued PVDF samples that remain free of Pt are examined with Raman spectroscopy to examine the relative phase content. The double side tape is used to attach the PVDF to the Si substrate. The PVDF thin films are analyzed by 532 nm green laser to observe PVDF Raman mode clearly. The Raman analysis is also carried out at room temperature to maintain the condition of fatigue tests. After 10^7^ bending, we observed that the atomic ordering of PVDF β-phase is maintained.

## 3. Results and Discussions

**Figure 2:**
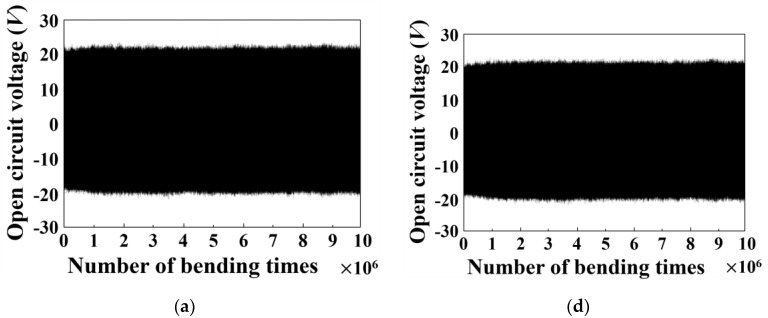

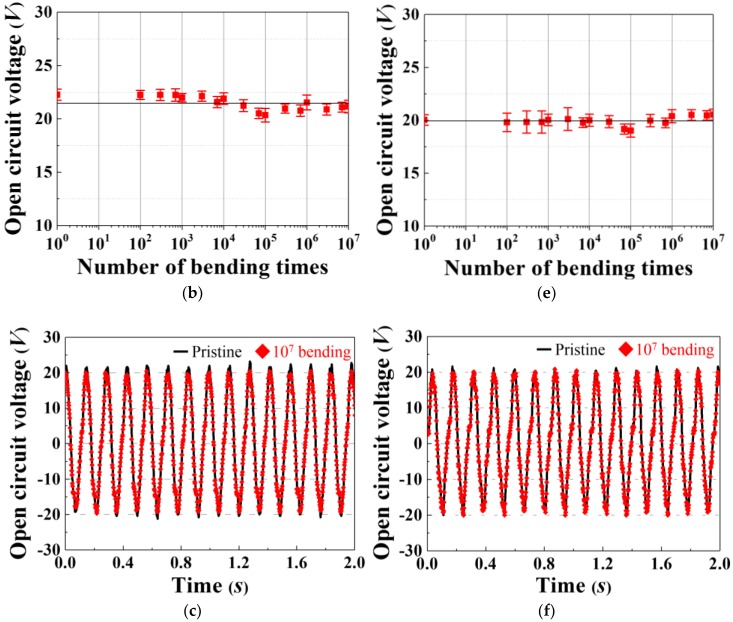
The measured open circuit voltages during repeated bending of 10^7^ times (**a**–**c**) The open circuit voltage profile for tensed PVDF layers (**d**–**f**) those for compressed PVDF layers.

Figure 2 shows the generated piezoelectric voltage for the tensed (Figure 2a–c) and the compressed (Figure 2d–f) PVDFs for the entire 10^7^ cycles. In Figure 2b,e, we plot the averaged peak voltages for 60 peaks around the specified bending number (10*^N^*) in logarithmic scale for better assessment of the fatigue behavior.

The relevant standard deviations are plotted as error bars in Figure 2b,e. For the tensed and compressed PVDF, the initial voltage output of 22.3 V and 20.0 V remain almost identical to 21.2 V and 20.5 V after 10^7^ cycles, respectively. These amazingly stable voltage measures indicate that the piezoelectric property of PVDF does not readily change upon applying repeated mechanical tension or compression, given the stable electrode adhesion. The voltage response to 7 Hz mechanical input frequency does not exhibit any notable change in phase behavior or voltage peak width either, as shown in Figure 2c,f.

**Figure 3:**
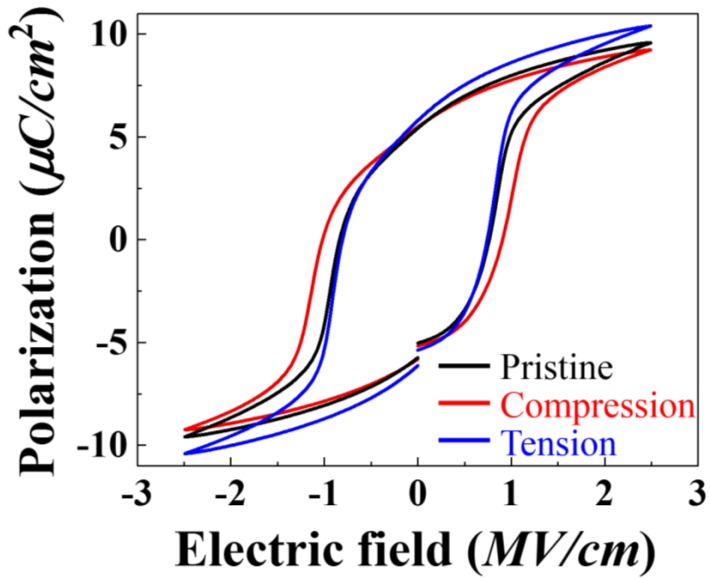
The PE hysteresis loop of PVDF at pristine and after applying 10^7^ tensile and compressive stressed. The initial remnant polarization of 5.48 μm/cm^2^ at pristine PVDF remains almost identical after tensile fatigue tests, however, decreases to 5.08 μm/cm^2^ compressive fatigue tests.

The polarization-electric field (PE) loops measured for the pristine and fatigued PVDF samples also reveal unaffected polarization properties of PVDF after repeated mechanical input. Figure 3 shows the polarization loops for the pristine, tensed and compressed PVDF. The remnant polarization of pristine PVDF is 5.48 μC/cm^2^, similar to previously reported corona-poled PVDF in β-phase [16,17]. Since the piezoelectric constants (d_ij_) are directly proportional to the remnant polarization values, assessing how fatigue affects the remnant polarization provides an indication of fatigue resistance against piezoelectricity loss.

The remnant polarization of PVDFs after 10^7^ cycles of repeated tension and compression are 5.51 μC/cm^2^ and 5.08 μC/cm^2^, respectively. These numbers are clearly far higher than the polarization loss observed by Zhu et al. after repeated bias application [6]. Interestingly, the compressed sample exhibits a notable decrease of 7.3% in remnant polarization. The saturation polarization also decreases by 4.2% from 9.5 μC/cm^2^ to 9.1 μC/cm^2^ for the compressed sample, while that of the tensed sample increases by 9.4% to 10.4 μC/cm^2^.

The anisotropic behavior of remnant polarization according to the stress direction calls for further investigation. While the decrease of 7.3% is not significant considering the excessive mechanical tests, the difference between tension and compression suggests that the fatigue resistance to de-poling may have an anisotropic response to the direction of stress. A potential explanation involves phase transformation into other polymorphs such as α- or γ-phase PVDF. Previous reports have shown that stretching a PVDF film results in α- to β-phase transformation [10]. While the strain is small in our fatigue tests compared to the stretch required for PVDF’s phase transformations, fatigue-induced phase transformations with small strains have been reported in various materials systems [18]. To examine whether indeed repeated mechanical strain results in different phase behavior, we examine the phase content of the pristine, tensed and compressed PVDF layers with Raman spectroscopy.

**Figure 4:**
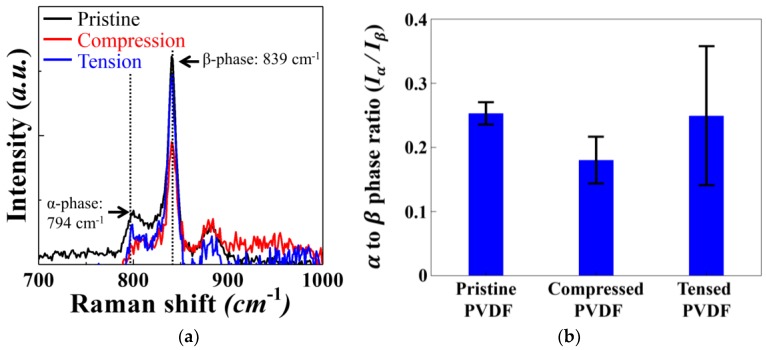
(**a**) The Raman shift observed for PVDF at pristine and after applying 10^7^ tensile and compressive stress. (**b**) The average intensity ratio (*I_α_/I_β_*) with the standard deviation plotted as the error bars.

Figure 4 plots the shift in Raman response for PVDF films. PVDF is known to possess phase-dependent characteristic Raman shifts: 839 cm^−1^ for the β-phase and 794 cm^−1^ for the α-phase [19]. We clearly observe the dominant β-phase in all three samples in Figure 4a, with notable peaks for the α-phase. Comparing the pristine sample with the compressed and tensed samples, we surprisingly observe increased α-phase content in tensed PVDF, and decreased α-phase content in compressed PVDF. Figure 4b plots the average intensity ratio (*I_α_/I_β_*) for four different pristine, compressed and tensed samples. The standard deviations are plotted as the error bars in the Figure 4b. Overall, the change is in the intensity is ratio is rather small after extensive mechanical tests. We also note that both the tensed and compressed samples exhibit increased error bar length compared to the pristine sample, with especially large deviation for the tensed sample. Although the results do not conclude that a clear anisotropic response to stress direction during mechanical fatigue exists, the increased deviation calls for further investigation into fatigue-induced phase transformation in PVDFs. Tracking the absolute amounts of the α-phase, β-phase and the non-crystalline phases may help us further clarify the effect of repeated mechanical stress on PVDF’s phase behavior. However, experimental difficulty exists for the current setup.

The stable piezoelectric properties of PVDF, demonstrated in this work by stable voltage output, remnant polarization and relative phase contents, not only suggests that PVDF possesses strong fatigue resistance against mechanical integrity loss or de-poling via domain switching. These results confirm the reports by Zhao et al. that the previously measured polarization loss during polarization fatigue likely results from the electrode delamination [7]. Our results also show that the electrode delamination may be easily engineered via using soft metal-based paste capped with the suitable sealing layer. As the material exhibits wide applicability ranging from piezoelectric generators or actuators to ferroelectric capacitors, its fundamental fatigue resistance calls for scientific understanding. Su et al. reported through first principles based calculations that nucleating nonpolar kinks in the PVDF chain (equivalent to seed for nonpolar phases) requires 24.8 kcal/mol [15]. In addition, the mobility of the polar-nonpolar interfaces requires the threshold tensile stress of approximately 2 GPa, a very high stress for soft PVDF with Young’s modulus of 2.9 GPa [20].

## 4. Conclusions

In summary, we report the excellent mechanical fatigue resistance of piezoelectric PVDF during 10^7^ cycles of mechanical loading. PVDF exhibits strong mechanical fatigue resistance against both tension and compression. The stable voltage output and remnant polarization suggest both little mechanical degradation such as cracking or pore formation and de-poling behavior such as domain switching. A 7% decrease in remnant polarization under repeated compression suggests a potential anisotropic response to stress directions. The repeated mechanical tests also result in deviations in phase behavior. The works demonstrate that PVDF-based piezoelectric energy harvesters or sensors possess suitable fatigue resistance for extended-life applications.
